# Plasma neutrophil extracellular trap level is modified by disease severity and inhaled corticosteroids in chronic inflammatory lung diseases

**DOI:** 10.1038/s41598-020-61253-2

**Published:** 2020-03-09

**Authors:** Zsófia Gál, András Gézsi, Éva Pállinger, Tamás Visnovitz, Adrienne Nagy, András Kiss, Monika Sultész, Zsuzsanna Csoma, Lilla Tamási, Gabriella Gálffy, Csaba Szalai

**Affiliations:** 1 Department of Genetics, Cell- and Immunobiology, Semmelweis University, Budapest, 1089 Hungary; 2 Heim Pál Children’s Hospital, Budapest, 1089 Hungary; 3 National Korányi Institute of TB and Pulmonology, Budapest, 1121 Hungary; 4 Department of Pulmonology, Semmelweis University, Budapest, 1083 Hungary; 5 Pulmonology Hospital Törökbálint, Törökbálint, 2045 Hungary; 6 MTA-SE Immune-Proteogenomics Extracellular Vesicle Research Group, Semmelweis University, Budapest, Hungary; 7 Department of Measurement and Information Systems, Budapest University of Technology and Economics, Budapest, Hungary

**Keywords:** Immunological disorders, Respiratory tract diseases, Cell biology, Immunology, Biomarkers, Diseases

## Abstract

A flow cytometry-based method was developed to quantify *in vivo* circulating neutrophil extracellular trap (NET) levels in plasma and compare them in patients with different chronic inflammatory lung diseases. Seventeen asthmatic and 11 control children, 12 adult controls, 46 asthmatic, 6 COPD and 6 adult patients with asthma-COPD overlap syndrome (ACOS) were recruited in the study. The presence of NETs in unstimulated cell-free plasma was confirmed and visualized by confocal laser-scanning microscopy. No significant differences were found in plasma NET levels between children and adults, children with or without asthma and adults with or without asthma, COPD or ACOS. When asthmatic patients were stratified according to their disease severity the average plasma NET level was significantly higher in asthmatic patients with more serious symptoms (adjusted p = 0.027). Patients with poorer pulmonary functions had higher plasma NET levels which negatively correlated with the FEV1 values (r = −0.39, p = 0.002). Patients who were medicated daily with inhaled corticosteroids (ICS) had significantly lower average plasma NET level than patients who did not or just occasionally used ICS (p = 0.027). If further studies confirm the NET-lowering effect of ICS in the circulation, it can be utilized in diseases where NETosis contributes to the pathogenesis.

## Introduction

Asthma is a complex chronic inflammatory disease accompanied by episodic airway obstruction and inflammation of the lower respiratory tract. As numerous triggers can provoke asthmatic symptoms, it is a heterogeneous condition especially in the type of inflammation^1^. Neutrophilic asthma with high neutrophil pulmonary infiltration is one of the most severe subgroup in asthma^2^. In contrast to asthma, patients with chronic obstructive pulmonary disease (COPD) have progressive and generally irreversible airflow obstruction. Disease progression, GOLD (Global Initiative for Chronic Obstructive Lung Disease) status and exacerbations of COPD are connected to neutrophilia in lungs^3–5^. Asthma-COPD overlap syndrome (ACOS) is a consensus-based phenotype, not an individual disease. In this condition, both asthmatic and COPD symptoms are present^6^.

Neutrophil granulocytes are the main cellular army of the innate immune system^7^. In the last decades, it has been discovered that neutrophils beside degranulation and phagocytosis can form neutrophil extracellular traps (NETs) to different triggers which process has been termed NETosis. *In vivo*, several biological molecules may elicit NETosis including interleukin-8 and tumor necrosis factor-α, both of which have a prominent role in asthma and COPD^3^. *In vitro*, the pharmacological agent phorbol-12-myristate-13-acetate (PMA) is a known strong inducer of NETosis that is routinely used in studies of NETs. NETs are generated through a signaling process that involves citrullination of histones by peptidylarginine deiminase 4 (PAD4) enzyme, chromatin decondensation, and disintegration of the nuclear membrane. NETs consist of chromatin complexes of cell-free DNA and citrullinated histones with attached neutrophil granular proteins (e.g. neutrophil elastase (NE), myeloperoxidase (MPO) and cathepsin G)^8^. NE is a serine protease which has virulence factor splitting and antibacterial impact^9,10^. MPO can produce hypochlorous acid which has a reactive antimicrobial effect^11^. NETs have an important role in host defense against invading pathogens by their sticky nature, steric inhibition and antimicrobial protein content^12^.

Recently, extracellular DNA traps have been identified in allergic asthmatic airways. NETs are increased mainly in neutrophilic asthma, but their contribution to disease severity is not clearly understood. The cause of NETosis in asthmatic airways is unknown. IL-8 can be a potential trigger of NETosis in the airways as it has been previously shown to induce NETosis^8,9^. If the content of NETs are permanent, induction of NETs are increased or clearance of traps are decreased, it can directly induce lung epithelial and endothelial cell death. Moreover, NETs might provoke airway remodeling and mucus hypersecretion^3^. NETosis can be a double-edged sword, as it helps to protect the host from pathogens, while released molecules can induce an inflammatory process causing tissue damage^13^. The deleterious effect of NETs has been investigated in many human conditions, infectious and non-infectious as well, including systemic lupus erythematosus, thrombosis, different types of cancer, diabetes mellitus, rheumatoid arthritis, acute respiratory distress syndrome, tuberculosis, cystic fibrosis and bacterial pneumonia^12,14,15^.

There are a few reports investigating NETosis in the airways of asthmatics, but until now there has been no study published about the plasma levels of NETs in patients with asthma, COPD or ACOS.

The quantification of NETosis is quite problematic. Visualization by fluorescence microscopy is subjective and not accurately quantifiable^16^. Most NET components are also released in soluble form from the neutrophils or during necrotic cell death, thus they are not NET-specific. The developed heterobifunctional ELISA methods can measure only two components at the same time. In the present study, to quantify the level of NETs in human platelet free plasma, in line with a paper of Gavillet *et al*., we have developed a flow cytometry-based method^17^. The method is based on parallel detection of cell-free DNA, citrullinated histone and MPO from cell-free plasma. The developed method was then used to detect and quantify *in vivo* circulating NETs in plasma from different patients. With flow cytometer targeting three different molecules in NET measurements can give more precise result and bias could also be avoidable.

The aim of the present study was to compare the plasma levels of NETs in children and adults with or without asthma, COPD or ACOS and to evaluate possible associations between the levels of NETs and different subgroups of patients.

## Results

### NET induction measured by flow cytometry-based method

**Figure 1:**
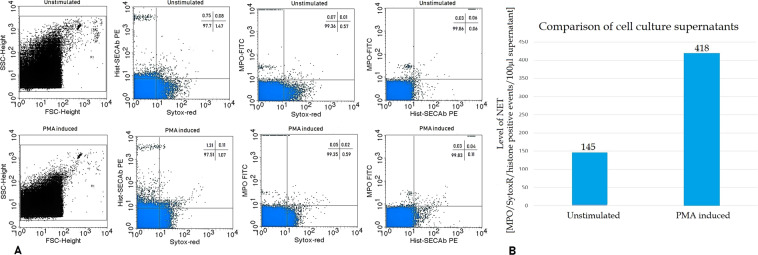
(**A**) Representative results of the flow cytometry measurement carried out in cell-free supernatants of the unstimulated (top row) and PMA induced neutrophil granulocytes. From left to right: FSC/SSC blot; Sytox Red (indicating free DNA)/histone H3; Sytox Red/MPO; histone H3/MPO double positive events. (**B**) Comparison of MPO/Sytox Red/histone H3 triple positive events in 100 µL PMA stimulated vs. unstimulated cell culture supernatants.

PMA induced isolated neutrophil granulocytes were used as positive controls for the setting of measurements with flow cytometer. The measurements were carried out in PMA treated and untreated cell-free supernatants. In Fig. 1A, a representative result of the flow cytometry measurement is shown. As can be seen in the figure, PMA treatment did not alter significantly the FSC/SSC dot blot and did not increase double positive events significantly either. On the other hand, the number of MPO/Sytox Red/histone triple positive events increased significantly in 100 µL cell culture supernatant (Fig. 1B).

### Visualization of NETs in plasma with confocal laser-scanning microscopy

With an independent method we also intended to confirm the presence of NETs in unstimulated plasma. There have been several published studies which visualized NETs from isolated and stimulated cells, however, according to our knowledge, not from cell-free plasma without induction. We intended to visualize NETs in plasma without NET induction by confocal laser-scanning microscope.

**Figure 2:**
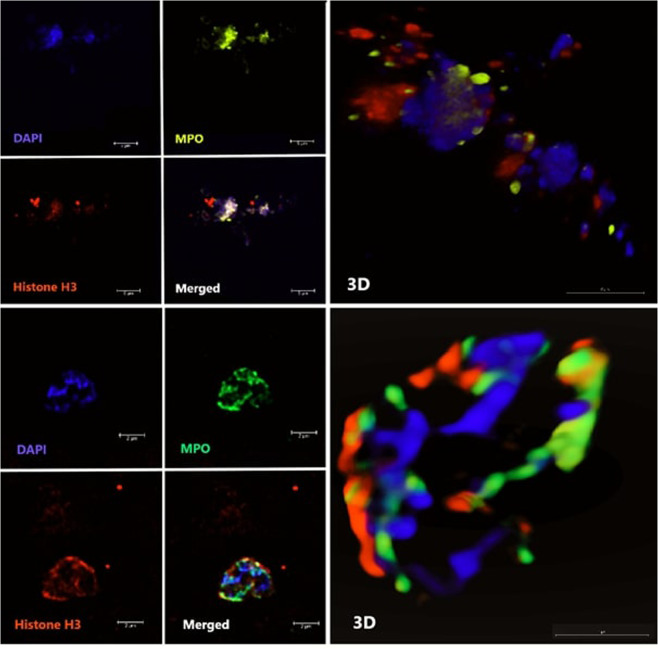
Two examples of *in vivo* circulating NET structures from unstimulated cell-free plasma, one from an asthmatic patient (upper photos) and one from a healthy control (bottom photos). Red coloured staining shows histone, blue DAPI indicates DNA presence, yellow and green coloured staining indicate MPO. The large, merged images show the structure of the NET. The 3D structure of the NETs can also be seen in a video in the Supplementary Material.

In Fig. 2 there are two examples of *in vivo* circulating NET structures from unstimulated cell-free plasma, one from an asthmatic patient and one from a healthy control. We considered the presence of a NET, where the colour labelled MPO, citrullinated histone and DNA overlapped. In the figure, red coloured staining shows histone, blue DAPI indicates DNA presence, yellow and green coloured staining indicate MPO. The 3D structure of the NETs can also be seen in a video in the Supplementary Material. According to these results the presence of NETs in unstimulated cell-free plasma was confirmed and visualized.

### Comparison of levels of NETs in plasma in human clinical samples

**Figure 3:**
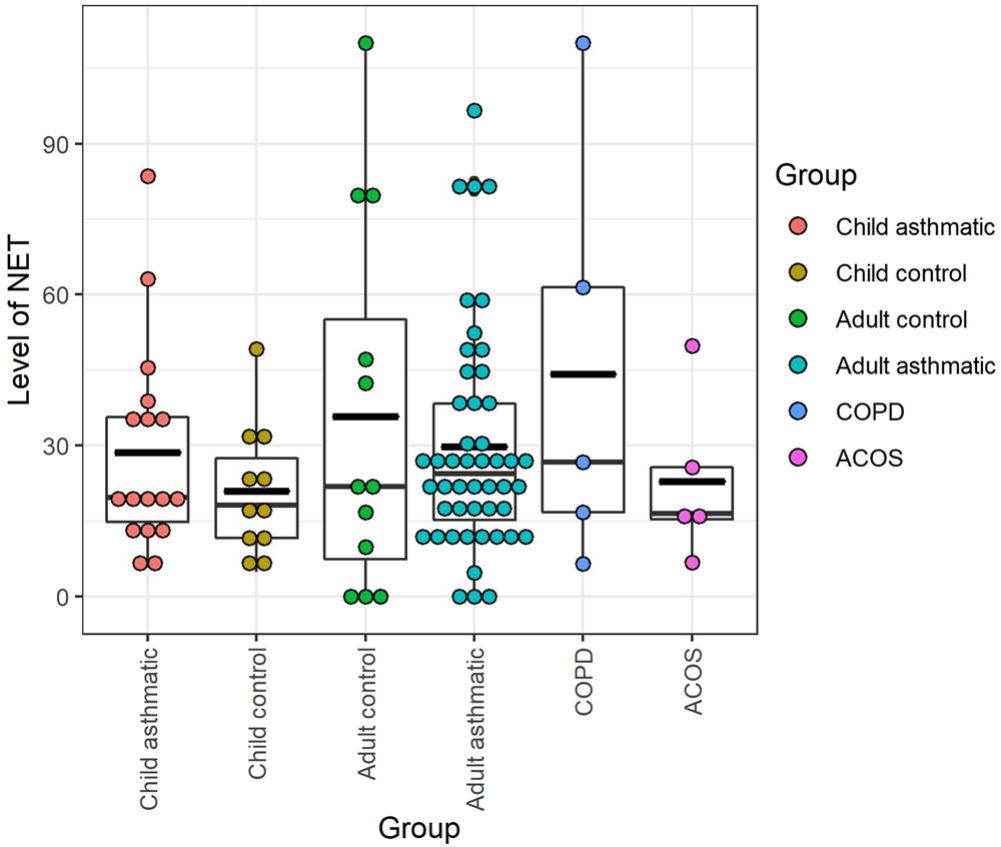
Comparison of *in vivo* plasma NET levels in different groups of patients. No significant differences were found in any comparisons.

*In vivo* circulating NETs were quantified and compared in 6 different groups of individuals: control and asthmatic children, control adults, and adult patients with asthma, COPD and ACOS. As can be seen in Fig. 3, the average levels of NETs did not differ significantly between these categories.

**Figure 4:**
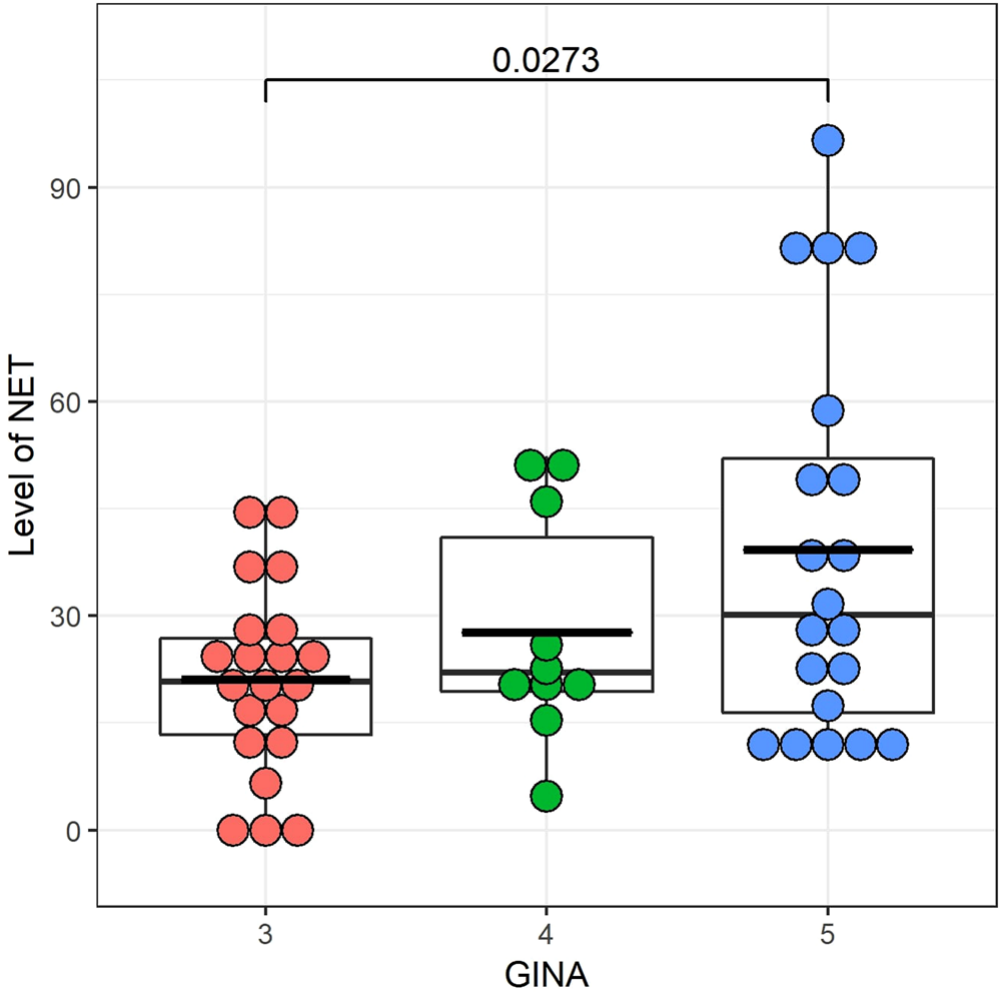
Comparison of the plasma NET levels in asthmatic patients stratified in different severity groups according to the Global Initiative for Asthma guidelines (www.ginasthma.org). The average NET levels in plasma increased parallel with the increasing severity and the difference was significant between GINA 3 and GINA 5 (unadjusted p = 0.013; adjusted p = 0.027).

Next, we investigated whether different asthma status or medication influenced the levels of NETs. According to GINA recommendations the asthmatic subjects were classified into categories based on the severity of their symptoms. In our population, there were 19 people with GINA 3, 8 with GINA 4 and 19 patients with GINA 5 (Table 1). We compared the average plasma levels of NETs between these severity groups. As can be seen in Fig. 4 the average NET levels in plasma increased parallel with the increasing severity and the difference was significant between GINA 3 and GINA 5 (adjusted p = 0.027).

**Table 1:** Clinical characteristics of the study population.

n	Children control	Asthmatic children^a^	Adult control	Asthmatic adult	COPD	ACOS
	11	17	12	46	6	6
age ± SD	9.4 ± 3.7	11.5 ± 3.9	35.1 ± 11.2	51.0 ± 13.2	68.3 ± 10.2	61 ± 7.6
gender(male/female)	10/1	13/4	4/8	14/32	2/4	4/2
**GINA status**^**b**^						
Number of patients in GINA 1(%)		7 (41)		1 (2)		
Number of patients in GINA 2(%)		6 (35)		2 (4)		
Number of patients in GINA 3(%)		4 (24)		15 (33)		
Number of patients in GINA 4(%)		0		8 (18)		
Number of patients in GINA 5(%)		0		19 (42)		
**FEV1(%)**^**c**^						
Value of FEV1 (<80%)		0		18	4	3
Value of FEV1 (>80%)		13		19	1	2
**Asthma characteristics**						
Controlled		15		10	1	1
Partly controlled		2		24	0	1
Uncontrolled		0		6	1	2
**Medical treatment**						
Occasionally medication with SABA (with or without low dose ICS)		8		21	0	2
Daily medication with ICS (alone or in combination)		6		16	3	3

COPD chronic obstructive pulmonary disease; ACOS asthma-COPD overlap syndrome; SD standard deviation; SABA short-acting β2-agonist; ICS inhaled corticosteroids.

^a^Asthmatic patients 18 years of age or under.

^b^Global Initiative for Asthma guidelines were applied to asthma diagnosis by physicians.

^c^FEV1(% of predicted): forced expiratory volume in one second measured by spirometry, as a part of pulmonary function tests.

**Figure 5:**
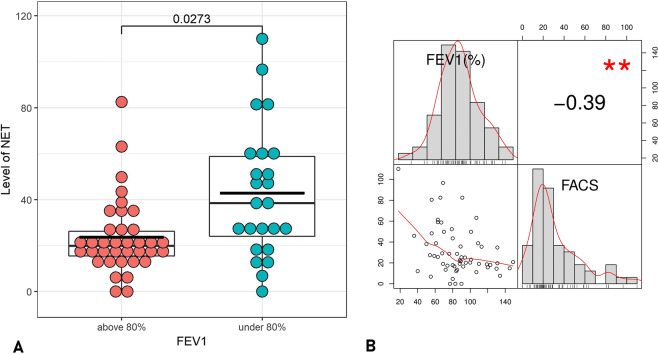
(**A**) Comparison of the plasma NET levels according to the FEV1 values of the patients. The adjusted p-value can be seen on the upper part of the figure (unadjusted p = 0.005). (**B**) Histograms of FEV1 (top left) and plasma NET levels (bottom right) with kernel density estimation (red lines), scatter plot between FEV1 and plasma NET levels (bottom left) with a fitted line, and Pearson’s correlation coefficient (top right) with ‘**’ indicating the significance of the relationship (p = 0.002).

According to their FEV1 (forced expiratory volume in one second) values, the patients were divided into two groups: FEV1 < 80% and FEV1 > 80%. As can be seen in Fig. 5A the average plasma NET level was significantly increased in patients with lower FEV1 than with higher FEV1 (adjusted p = 0.027). As FEV1 is a continuous variable, we also calculated Pearson’s correlation coefficient between FEV1 and plasma NET levels. As presented in Fig. 5B there was a statistically significant negative correlation (r = −0.39, p = 0.002) between the plasma NET levels and the FEV1 values.

### Daily inhaled corticosteroid associates with lower plasma NETs

We also investigated whether medication might influence the plasma NET level in the patients. We categorized the patients into two groups: (1) patients who occasionally used short-acting beta-agonists (SABA) with or without low dose inhaled corticosteroid (ICS) as needed; (2) patients who were continuously (daily) medicated with ICS (alone or in combination with long-acting beta agonist (LABA), long-acting muscarinic receptor antagonist (LAMA), leukotriene receptor antagonist (LTRA) or theophylline) some of them occasionally also used SABA.

**Figure 6:**
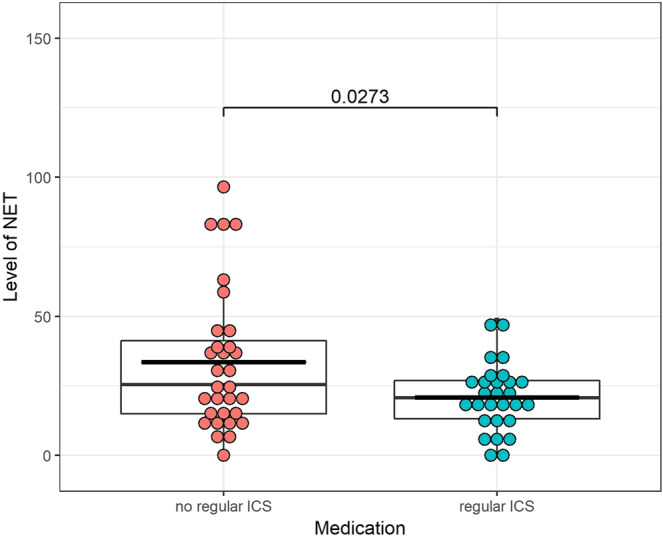
Comparison of the plasma NET levels in patients stratified according to use of inhaled corticosteroids. The group on the left involves patient who occasionally used SABA with or without low dose ICS as needed; the group on the right consists of patients who used ICS daily. The adjusted p-value can be seen on the upper part of the figure (unadjusted p = 0.016).

As can be seen in Fig. 6, those patients who took ICS daily had significantly lower average plasma NET level than those who did not (unadjusted p = 0.016, adjusted p = 0.027).

## Discussion

In the present study we developed a flow cytometry-based method to quantify neutrophil extracellular traps in unstimulated cell-free plasma and visualized them with confocal laser-scanning microscopy. We found no significant differences in average plasma NET levels between different chronic lung diseases but disease severity and regular ICS usage seemed to influence them.

Earlier it was shown that extracellular traps were generated in human atopic asthmatic airways *in vivo* measured in endobronchial biopsy specimens^18^. Later it was also demonstrated that components of NET were significantly elevated in sputum of patients with asthma and COPD compared with healthy controls^19^. According to our results, however, there was no significant difference between plasma NET levels in controls and patients with different chronic lung diseases. The results also indicated that a certain level of NETosis could also be measured in healthy individuals, in some of them in a relatively high level. This may suggest that e.g. silent infection can activate the innate immune system including NETosis which may eliminate or control the infectious agents without causing symptoms.

There are several diseases which associate with elevated serum or plasma NET levels, including diabetes, vasculitis, thrombosis or SLE, and in these diseases NETs probably also participate in the pathogenesis^14,20,21^. In all of these diseases the inflammation or the processes leading to NETosis take place in the blood vessels causing elevated blood NET levels. It seems, that in chronic airway diseases the elevated NETosis happens mainly in the airways whose effect, apparently, cannot be detected in the circulation.

Interestingly, however, when the patients were stratified into different disease subgroups there were significant differences between certain groups in this respect. Higher plasma NET levels were detected in groups of patients with more serious diseases relative to groups of patients with less severe symptoms. The plasma NET levels negatively correlated with FEV1 values. These might be explained by the observations that impaired lung function associates with a low grade of systemic inflammation with an elevated plasma CRP level. In addition, it was also demonstrated that plasma CRP were inversely associated with FEV1^22^. Furthermore, it was shown that CRP induces a concentration-dependent NET synthesis^23^. A possible hypothesis can be that impaired lung function leads to a systemic inflammation with elevated CRP level which induces NETosis. Presently, however, there is a controversy in the scientific literature whether increased systemic inflammation leads to impaired lung function or inversely^24^. It also requires further studies, whether this elevated NET level contributes to the impaired lung function or to the disease pathogenesis creating a vicious circle.

To the best of our knowledge, no study has investigated NET formation *in vivo* during treatment using inhaled corticosteroids in humans. According to our results, the average plasma NET level was significantly lower in patients treated daily with ICS than in patients who used it occasionally or not at all. Earlier, PMA induced NETs were found to be sensitive to non-steroidal anti-inflammatory drugs *in vitro* but were reported to be unaffected by glucocorticoids (GC)^25^. However, in an equine model of asthma Varga S. *et al*. showed that GC decreased PMA-induced NETs formation *in vitro* and also *in vivo* in the lungs of severe asthmatic horses^26^. In another study, a marked reduction of reactive oxygen species generation was observed in neutrophils following intravenous administration of dexamethasone in human healthy subjects^27^. Based on these observations, presently it cannot be explained why patients treated daily with ICS have lower plasma NET levels. In our study, however, contrary to the previous studies we measured unstimulated, *in vivo* circulating NETs in cell-free plasma. Furthermore, ICS are absorbed into the systemic circulation either by deposition in the oropharynx where they are swallowed, or by deposition in pulmonary airways^28^. It is well known that NETosis is dependent on the production of reactive oxygen species^29^. As corticosteroids inhibit reactive oxygen species generation, the absorbed ICS in the circulation can also inhibit NETosis, causing lower levels of NETs. Naturally, further studies are needed to prove this hypothesis.

In our study we used a modified version of the flow cytometric method developed by Gavillet *et al*.^17^ where the measurement was performed in blood cell suspension, while we measured cell-free plasma utilizing our previous experiences in extracellular vesicle quantification^30–32^. Gavillet *et al*. detected histone H3 and MPO double positive particles, we counted MPO/free DNA/histone H3 triple positive events. Using PMA induced isolated neutrophil granulocytes we found larger differences in triple positive than in double positive events between the supernatants of induced and non-induced neutrophils. We could also verify the presence of *in vivo* circulating NETs in cell-free plasma by confocal laser-scanning microscopy visualizing events where the colour labelled MPO, citrullinated histone and DNA overlapped.

Here, we have to mention some weaknesses of our study. We did not have the blood neutrophil and eosinophil levels from all of our patients and accordingly we did not use these data in our analyses. Our results, however, suggest that the measured plasma NET levels are probably the results of processes in the circulation and earlier it was shown that the blood neutrophil levels did not differ significantly between different types of asthma (neutrophilic vs. eosinophilic vs. paucigranulocytic, etc.)^33^. The blood neutrophil levels were found to be higher in patients with COPD relative to controls^34^, but in our study we did not find differences in plasma NET levels. Another weakness is the relative few patients, especially in COPD and ACOS. But, based on the results presented in Fig. 3, it has a low probability, although it cannot be excluded, that the differences would be clinically relevant in larger groups of patients. We also have to note that we confirmed our results only with the semi-quantitative confocal laser-scanning microscopy, where we used also three different NET markers. The presently available quantitative heterobifunctional ELISA methods, however, can measure only two parameters and when we measured two targets with flow cytometer, differences could be barely found between unstimulated and PMA induced samples (Fig. 1A). On the other hand, the number of MPO/Sytox Red/histone triple positive events increased significantly (Fig. 1B).

In conclusion, we have developed a flow cytometry-based method to quantify *in vivo* circulating NETs in plasma. We could visualize for the first time *in vivo* circulating NETs in cell-free plasma. We did not find significant differences in average plasma NET levels between children and adults, children with or without asthma and adults with or without asthma, COPD or ACOS. We found higher average plasma NET level in asthmatic patients with GINA 5 comparing them to patients with GINA 3; and there was an inverse correlation between FEV1 and plasma NET levels. According to our results, plasma NET levels were lower in patients who regularly used ICS than in patients who did not.

The developed flow cytometry-based method might be used in clinical settings and thus offer novel possibilities of clinical and biological investigations for detecting *in vivo* circulating NETs as a biomarker. If further studies confirm the NET-lowering effect of ICS in the circulation, it can be utilized in diseases where NETosis contributes to the pathogenesis.

## Methods

### Participants

Participants with asthma, COPD and ACOS were recruited from Asthma ambulance of National Korányi Institute of TB and Pulmonology (n = 32), Allergology Department of Heim Pál Children’s Hospital (n = 17), Department of Pulmonology of Semmelweis University (n = 26). Asthmatic subjects (n = 63) were diagnosed based on Global Initiative for Asthma guidelines (www.ginasthma.org), as we have written previously^35^. COPD (n = 6) diagnosis was determined according to the Global Initiative for Obstructive Lung Diseases (https://goldcopd.org) designation. Asthma control status was determined by the physicians treating the patients as described in elsewhere^36–38^. In case of ACOS (n = 6), where patients show both asthmatic and COPD symptoms we followed GINA and GOLD strategy^4,5^. The control group consisted of 23 individuals with no history of asthma or allergy, children (n = 11) from the Department of Urology and Department of Ear, Nose and Throat Medicine of Heim Pál Hospital. Control adults (n = 12) were healthy donors. Subjects were all Caucasian with about 5% Gypsy origin based on Hungarian statistical databases. Detailed information of the study population can be found in Table 1.

Written informed consent was provided by all participants or parent/guardian at the time of recruitment. This case-control study was conducted according to the principles determined in the Declaration of Helsinki and the whole study including the informed consent procedure were approved by the Hungarian Scientific and Research Ethics Committee of the Medical Research Council (ETT TUKEB; Case No.: 3526-0/2010-1018EKU; 14666-1/2012/EKU; IF-980-9/2016).

### Neutrophil isolation and flow cytometry-based method to detect NETs

For the method development, PMA induced isolated neutrophil granulocytes were used as positive controls in measurements with flow cytometer. Neutrophil preparation was conducted using density gradient method with Dextran (5%) and Percoll (63% and 72%) solutions starting from EDTA blood collection tube^39^. Induction of isolated neutrophils were carried out as it has been written elsewhere^40^. PMA (100×) and 2% BSA (bovine serum albumin) were added to the separated granulocytes. Four-hour incubation was implemented at 37 degrees Celsius. After centrifugation, supernatant was treated with antibodies and DNA stain as described below.

NETs were determined by anti-MPO FITC (anti-human mouse IgG, BD Biosciences, San Jose, CA, USA), rabbit anti-histone H3 (citrulline R2 + R8 + R17) antibody (ab5103, Abcam, Cambridge, UK) and SYTOX Red dead cell stain (Invitrogen, Thermo Fischer Scientific, Waltham, MA, USA), which is a high-affinity nucleic acid stain. As anti-histone H3 antibody does not have fluorescent label, secondary antibody was needed (rabbit IgG PE-conjugated antibody, R&D Systems, Minneapolis, MN, USA). All the above mentioned antibodies were used in 1:700 dilution. Samples and the first three mentioned labels were pipetted to a FACS tube with 4% 0.22 µm filtered paraformaldehyde (PFA). After 15-minute incubation in dark, secondary antibody was added to the solution, following an additional 15 minutes incubation in dark. Filtered 1% PBS solution was added to the tube with PKH26 Reference Microbeads (Sigma-Aldrich, Merck, Darmstadt, Germany). To avoid the bias from aspecific binding of the secondary antibody we used the secondary antibody and samples without the other three labels as a background. Measurements were conducted by BD FACSCalibur (BD Biosciences). Optimization of cytometer settings and gating strategy was carried out as described earlier for extracellular vesicle quantification^30–32^. For the quantification only the number of MPO/Sytox Red/histone triple positive events were considered.

### Human samples

Whole blood samples were collected to sodium citrate tubes from all individuals. During the blood collection no patients were in exacerbation. Platelet free plasma samples were prepared by centrifugation two times at 2500 RPM for 15 minutes. For *in vivo* NET quantification the platelet free plasma samples were processed as described above.

### Confocal laser-scanning microscopy

Platelet free plasma samples (10 µL) were attached to MilliCell EZ slides (Merck-Millipore), dried for 10 minutes at room temperature (RT) and were fixed with 4% 0.22 µm filtered PFA for 20 minutes at RT. Following three washes with 1% PBS, samples were blocked with 5% BSA and incubated for 30 minutes at RT. Because of NETs occurrence we used BSA instead of fetal bovine serum (FBS). Primary antibodies (mouse monoclonal anti-MPO FITC (BD Biosciences) and rabbit polyclonal anti-histone H3 antibody (ab5103, Abcam) in 5% BSA solution, both same as before) were used in 1:100 dilution for 1 hour at RT in dark. After three times washes with 5% BSA, secondary antibodies were applied such as F(ab’)2-goat anti-mouse IgG (H + L) conjugated with eFluor 570 (eBioscience) in 1:200 dilution and goat anti-rabbit IgG conjugated with ATTO647N (Sigma-Aldrich) in 1:500 dilution for 1 hour at RT in dark. Washed twice with 1% PBS and twice with distillated water. Slides were mounted with DAPI containing Prolong Diamond (Invitrogen). Samples were examined by Lecia SP8 laser-confocal microscope (Leica Microsystems, Wetzlar, Germany).

### Statistical analysis

All statistical evaluations were performed with R statistical computing program (R Foundation for Statistical Computing, Vienna, Austria). Differences between experimental groups were analysed by one-way ANOVA. Differences between subgroups of patients were analysed by Student’s t-test (unpaired, two-tailed). P-values were adjusted with Benjamini Hochberg method to correct for the false discovery rate, and adjusted p-values <0.05 were considered to be statistically significant. The relationship between the level of NET and FEV1 was assessed by calculating Pearson’s correlation coefficient. R statistical computing program was used for graphic illustrations.

## Supplementary information

Supplementary information.

Supplementary information 2.

Supplementary Information 3

## Data Availability

All datasets and protocols of the measurements are available in request.
