# Author Correction: Plasma neutrophil extracellular trap level is modified by disease severity and inhaled corticosteroids in chronic inflammatory lung diseases

**DOI:** 10.1038/s41598-020-69873-4

**Published:** 2020-07-31

**Authors:** Zsófia Gál, András Gézsi, Éva pállinger, Tamás Visnovitz, Adrienne Nagy, András Kiss, Monika Sultész, Zsuzsanna csoma, Lilla tamási, Gabriella Gálffy, Csaba Szalai

**Affiliations:** 1 Department of Genetics, Cell- and Immunobiology, Semmelweis University, Budapest, 1089 Hungary; 2 Heim Pál Children’s Hospital, Budapest, 1089 Hungary; 3 National Korányi Institute of TB and Pulmonology, Budapest, 1121 Hungary; 4 Department of Pulmonology, Semmelweis University, Budapest, 1083 Hungary; 5 Pulmonology Hospital Törökbálint, Törökbálint, 2045 Hungary; 6 MTA-SE Immune-Proteogenomics Extracellular Vesicle Research Group, Semmelweis University, Budapest, Hungary; 7 Department of Measurement and Information Systems, Budapest University of Technology and Economics, Budapest, Hungary

Correction to: *Scientific Reports*https://doi.org/10.1038/s41598-020-61253-2, published online 09 March 2020

This Article contained errors, whereby two affiliations were omitted for the author András Gézsi. The correct affiliations for András Gézsi are listed below:

Department of Genetics, Cell- and Immunobiology, Semmelweis University, Budapest, 1089, Hungary

MTA-SE Immune-Proteogenomics Extracellular Vesicle Research Group, Semmelweis University, Budapest, Hungary

Department of Measurement and Information Systems, Budapest University of Technology and Economics, Budapest, Hungary

In addition, the Figure Legend for Supplementary Videos 1 and 2 were omitted from the Supplementary Information 3 file. The correct legend for these videos can be seen below:

“Two video presentations of the 3D structures of the in vivo circulating NETs from unstimulated cell-free plasma shown in figure 2.”

These errors have now been corrected in the HTML and PDF versions of this Article, and in the accompanying Supplemental Information 3 file.

