# Supplementary figures and images for: Plasma neutrophil extracellular trap level is modified by disease severity and inhaled corticosteroids in chronic inflammatory lung diseases
Source: Sci Rep. 2020 Mar 9;10:4320. doi: 10.1038/s41598-020-61253-2 (PMC7062787; doi:10.1038/s41598-020-61253-2)

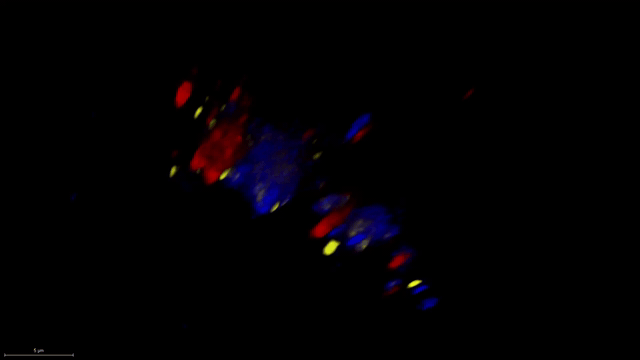

Supplement: Supplementary file 1 — Supplementary information. [file 41598_2020_61253_MOESM1_ESM.gif]

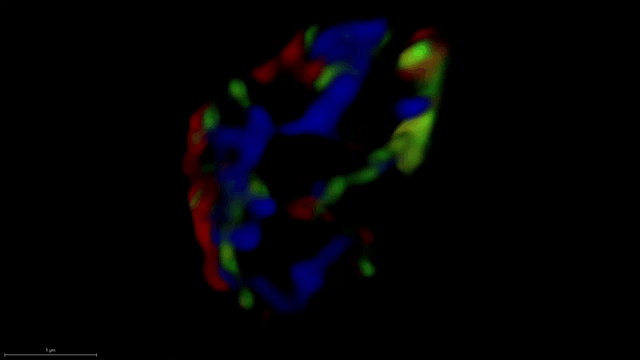

Supplement: Supplementary file 2 — Supplementary information 2. [file 41598_2020_61253_MOESM2_ESM.gif]
