# Supplementary material for: Plasma neutrophil extracellular trap level is modified by disease severity and inhaled corticosteroids in chronic inflammatory lung diseases
Source: Sci Rep. 2020 Mar 9;10:4320. doi: 10.1038/s41598-020-61253-2 (PMC7062787; doi:10.1038/s41598-020-61253-2)
Supplement: Supplementary file 3 — Supplementary Information 3 [file 41598_2020_61253_MOESM3_ESM.docx]

**Plasma neutrophil extracellular trap level is modified by disease severity and inhaled corticosteroids in chronic inflammatory lung diseases**

**Authors**

Zsófia Gál^1^, András Gézsi^1,6,7^, Éva Pállinger^1^, Tamás Visnovitz^1^, Adrienne Nagy^2^, András Kiss^2^, Mónika Sultész^2^, Zsuzsanna Csoma^3^, Lilla Tamási^4^, Gabriella Gálffy^5^, Csaba Szalai^1,2^*

**Author Affiliations**

^1^Department of Genetics, Cell- and Immunobiology, Semmelweis University, Budapest, 1089 Hungary

^2^Heim Pál Children’s Hospital, Budapest, 1089, Hungary

^3^National Korányi Institute of TB and Pulmonology, Budapest, 1121, Hungary

^4^Department of Pulmonology, Semmelweis University, Budapest, 1083, Hungary

^5^ Pulmonology Hospital Törökbálint, Törökbálint, 2045, Hungary

^6^ MTA-SE Immune-Proteogenomics Extracellular Vesicle Research Group, Semmelweis University, Budapest, Hungary

^7^ Department of Measurement and Information Systems, Budapest University of Technology and Economics, Budapest, Hungary

***Correspondence, proofs and reprints:**

Csaba Szalai, PhD, DSc; Department of Genetics, Cell- and Immunobiology, Semmelweis University, Budapest, Hungary; H-1089 Nagyvárad tér 4. Telephone: +36-1-210-2930/56236; Fax: +36-1-303 6968 Email: szalaics@gmail.com

**Supplementary Video Legend**

Two video presentations of the 3D structures of the *in vivo* circulating NETs from unstimulated cell-free plasma shown in figure 2.
